# Motor skills at 7 years of age and spinal pain at 11 years of age: a cohort study of 26,000 preadolescents

**DOI:** 10.1007/s00431-023-04964-8

**Published:** 2023-04-12

**Authors:** L. Hestbæk, S. J. Kamper, J. Hartvigsen, A. C. Falch-Joergensen

**Affiliations:** 1 The Chiropractic Knowledge Hub, Campusvej 55, 5230 Odense, Denmark; 2 Dept. of Sports Science and Clinical Biomechanics, University of Southern Denmark, Odense, Denmark; 3 University of Sydney & Nepean Blue Mountains Local Health District, Sydney, Australia; 4 Section of Epidemiology, Department of Public Health, Faculty of Health and Medical Science, University of Copenhagen, Copenhagen, Denmark

**Keywords:** Children, Back pain, Neck pain, Motor skills, Birth cohort

## Abstract

This study aims to investigate the relationship between motor skills at age 7 and spinal pain at age 11. The study included participants from the Danish National Birth Cohort. Data on motor skills were obtained from the Developmental Coordination Disorder Questionnaire, completed by the mothers when the children were 7 years old, and spinal pain was self-reported at age 11 for frequency and intensity of neck, mid back, and low back pain. This was categorized into “no,” “moderate,” or “severe” pain, based on frequency and pain intensity. Associations were estimated using multinomial logistic regression models. Data on both motor skills and spinal pain was available for 25,000 children. There was a consistent pattern of reporting more neck or mid back pain at age 11 for those with lower levels of fine motor skills and coordination scores at age 11. The relationship was significant for severe pain (the highest relative risk ratio being 1.87 and the lowest 1.18), but not for moderate pain (the highest relative risk ratio being 1.22 and the lowest 1.07). Gross motor skills were not associated with spinal pain, and there was no relationship between low back pain and motor skills.

*  Conclusion*: Our results indicate a link between motor development at 7 years of age and neck and mid back pain, but not low back pain, at 11 years of age. Improvement of motor skills in young children might reduce the future burden of neck and mid back pain and should be a target of future research.

**Table:** 

**What is Known:**
• *Spinal pain in preadolescence and adolescence is common and predisposes to spinal pain in adulthood.*
• *Motor skills influence the biomechanics of movement and therefore has a potential impact on musculoskeletal health.*
**What is New:**
• *Poor fine motor- and coordination skills in childhood were associated with increased risk of severe neck- or mid back pain, but not low back pain, four 4 years later.*
• *Poor gross motor skills were not associated with higher risk of later spinal pain.*

**Supplementary Information:**

The online version contains supplementary material available at 10.1007/s00431-023-04964-8.

## Introduction

Back and neck pain are major contributors to the global burden of disease [1] and the most common somatic reasons for disability pension in Scandinavia [2, 3]. It is often considered as a problem relating to the working age population, but research has shown that it can have its onset already early in life [4–6], that prevalence approaches adult levels around the age of 18 [7, 8], and that teenagers with back pain are likely to become adults with back pain [9]. Therefore, it is essential to identify risk factors predisposing to spinal pain early in life to improve our chances for prevention [5].

Good motor skills are considered important for children’s physical, social, and psychological development. Motor skills are also important for an active lifestyle, since several studies have shown a positive association between good motor skills and higher levels of physical activity [10–12]. Consequently, there is evidence of many health benefits to be gained from an improvement in motor skills. For instance, it has been demonstrated that good motor skills positively influence cardiorespiratory fitness [10, 13], body weight [10, 14–16], and sports participation [10, 16], all suggesting that early competency in motor skills may have important health implications.

There is also reason to believe that motor skills may be associated with musculoskeletal problems as motor skills are the ability to perform certain tasks such as walking or catching a ball, which in turn is highly dependent on both balance (the ability to stay upright or stay in control of body movement) and coordination (the ability to move two or more body parts under control, smoothly, and effectively). The level of motor skills strongly influences motor performance, i.e., the ability to perform tasks involving movement. Motor performance in children with Developmental Coordination Disorder (DCD) is usually slower, less accurate, and more variable than in their peers. Motor learning is also impacted, with children with DCD having difficulty acquiring skills typically learned during childhood, such as tying shoes or riding a bicycle [17]. Physical education can also be affected, as children with DCD have trouble throwing, catching, or kicking a ball, running, skipping, and playing sports [17]. Age-appropriate balance and coordination allows the child to be involved in sports participation with a reasonable degree of success as it aids fluid body movement for physical skill performance (e.g. walking a balance beam or playing football), and therefore, it is likely that both quantity and quality of physical activity are affected in children with poor coordination, even if they do not meet the criteria for DCD. With good balance and coordination, there is less likelihood of injury as the child is likely to have appropriate postural responses when needed (e.g. putting hands out to protect themselves when falling of a bike). The physical attributes of balance and coordination also allow appropriate biomechanical function of the musculoskeletal system and thus reduces the risk of inappropriate load of joints.

Furthermore, children with DCD engage in fewer physical and group activities than their peers, perhaps as a reflection of their poorer athletic performance and social competence, and this can lead to social isolation [17], which has also been observed in children with spinal pain [4, 18, 19]. Again, as levels of coordination and motor skills are based on a continuum, is it reasonable to assume that the same mechanisms are present in children with a lesser degree of coordination impairment, i.e., not reaching the threshold for DCD.

The presence of a link between spinal pain and motor coordination and/or motor skills is supported by intervention studies, which have shown that focused motor skills training can increase muscle control, coordination, and balance in adults and decrease back pain–related disability compared to other types of exercise [20, 21]. Good motor control has also been shown to reduce the frequency of musculoskeletal injuries in the extremities [22–24], which again might help to reduce spinal pain, as two studies have shown an increased occurrence of spinal pain in case of lower extremity pain [25, 26].

Given that motor delay observed in childhood are still apparent in adolescence [27], it is possible that development of motor skills early in life may influence the onset, or the maintenance, of spinal pain in childhood. Our group has previously conducted a large study of motor mile stones and found no association between age of unsupported sitting and independent walking and later spinal pain [28]. However, a link between motor coordination in childhood and the development of back and neck pain has not been investigated.

This study is an extension of the previous study [28], taking advantage of the extensive database in the Danish National Birth Cohort to investigate a potential relationship between motor development in childhood and spinal pain in preadolescence. Specifically, we estimated the magnitude of the relationship between motor coordination at 7 years of age and the presence of spinal pain (SP) at 11 years of age.

## Material and methods

### Population and data collection

The Danish National Birth Cohort (DNBC) began as a survey of pregnant women in Denmark. Between 1996 and 2002, pregnant women were invited to enrol in the cohort at their first antenatal visit to the general practitioner. Women were included if they intended to carry the pregnancy to term and spoke Danish well enough to complete telephone interviews. Assessment of participants was conducted with interview surveys of the mothers, while the participants were in utero and again at the ages of 6 and 18 months, as well as maternal questionnaires when the children were around 7 years of age. Surveys included a range of socio-demographic, anthropometric, health-related, and behavioural variables of both mothers and children as well as motor development.

A follow-up was planned for the year the children turned 11, where a questionnaire was distributed by mail and completed by the children themselves. However, administration of the questionnaires was somewhat irregular, and therefore, the age ranges from 10 to 14, but with the majority being 11 years of age at the time of questionnaire completion (henceforth called age 11 for readability). The questionnaire included data on spinal pain.

The target sample for the analyses in this study consisted of liveborn singletons included in the DNBC. Children were included in the analyses if their mothers had completed the 7-year survey, including questions about motor skills, and the children themselves had answered the questions about spinal pain at age 11. However, if children have serious physical or developmental problems, they are very likely to experience developmental delay as well as later spinal pain, and thus, a potential relationship could be driven by these children. Therefore, children were excluded from the analyses if their mothers answered positive to the question “The following questions are about what your child can do right now, but first I need to know if he/she has any serious physical or developmental problems? “ at the 18-month interview.

The Danish National Birth Cohort is presented in detail elsewhere [29].

### Variables

#### Exposure: motor development at seven years of age (questionnaire completed by the mother)

The Developmental Coordination Disorder Questionnaire (DCDQ) was used to assess motor development at age seven. Parents were asked to assess 15 statements related to their child as compared to other children of the same age and sex. Each question had five response options: (1) “not true,” (2) “a little true,” (3) “fairly true,” (4) “true,” and (5) “very true,” giving a maximum score of 75. The scoring is divided into subscales for three domains: statements 1–6 (max 30 points) evaluating “control during movement,” statements 7–10 (max 20 points) evaluating “fine motor/handwriting,” and statements 11–15 (max 25 points) about “general coordination.”

The DCDQ was developed to screen for Developmental Coordination Disorder [30] and has been validated for use with 7-year-old children [31, 32].

#### Outcome: spinal pain (questionnaire completed by the child at age 11)

The Young Spine Questionnaire (YSQ) includes assessment of presence, frequency, and intensity of neck pain, mid back pain, and low back pain. The YSQ was developed for and tested among 5–12-years old and is described elsewhere [33]. The exact wording of the frequency questions was “How often have you had pain in your neck/middle back/low back,” with four response categories: “often”, “once in a while,” “once or twice,” and “never.” Respondents that had experienced pain were requested to rate the pain intensity by means of The Revised Faces Pain Scale (rFPS), ranging from 1 (“Not at all”) to 6 (“Really very much”).

**Fig. 1:**
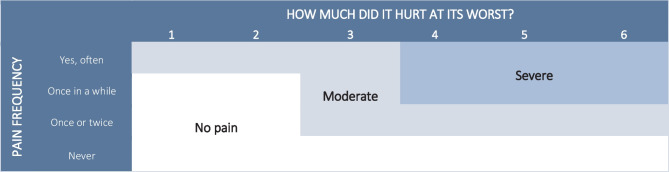
Definition of “no pain,” “moderate pain,” and “severe pain,” based on frequency and intensity of pain

To distinguish between trivial and non-trivial pain, we combined pain frequency and intensity for each spinal region into “no pain,” “moderate pain,” or “severe pain,” in the same manner as in a previous study of the same cohort [34]. The optimal cut-point for consequential spinal pain in children is presently unknown but based on findings from previous studies of children in this age group [35]; also, using the YSQ, severe pain was defined as pain of four or more on the Faces Pain Scale-Revised [36] and occurring at least “once in a while.” No pain was defined as “never” or “once or twice”/”once in a while” with pain intensity below 3. Exact classification of pain groups appears from Fig. 1. Overall spinal pain was defined as a composite variable including the three spinal regions. If the reported pain differed between the three spinal locations, the location with the most severe pain was used.

The primary outcomes are moderate and severe spinal pain at 11 years of age, and secondary outcomes are pain in the three areas of the spine separately, i.e., neck pain, mid back pain, and low back pain.

#### Confounders

In the previous study, investigating the relationship between motor milestones and later spinal pain [28], sex, birthweight, maternal smoking during pregnancy, maternal alcohol consumption during pregnancy, household income, and mother’s level of schooling (20 response options categorized into four levels [37]) were found to be confounders for at least one spinal region. Therefore, they were a priori selected to be included in the present analyses.

Furthermore, overweight or excess body fat has been found to increase the risk of both poor motor skills [38] and spinal pain, also in children [39], and therefore, height and weight at baseline were also included as potential confounders.

All of these confounders were reported by the mother at various follow-up times, except birth weight, which was obtained from the Danish Medical Birth Register [40].

For the exact wording of questions and response options, see Supplementary Material 1.

### Analyses

To assess representativeness, the study samples for univariate and adjusted analyses were compared to the original cohort with respect to exposure, outcomes, and confounders.

Continuous data were reported as means with standard deviations (SD) and dichotomous and categorical data as absolute numbers and proportions.

At 7 years of age, the DCDQ scores were very skewed with a marked ceiling effect. Therefore, based on visual inspection of the distribution, the total score and all subscales were divided into three categories for interpretation:

- 3 = the lowest 10% (poorest development of motor skills) which is the validated cut-point for DCD [32].
- 2 = above the tenth percentile but below maximum score.
- 1 = maximum score (best development of motor skills).

The relationship between motor skills and later spinal pain was depicted graphically by showing the prevalence of *all* spinal pain (moderate and severe combined) by motor skills categories before estimates of associations were calculated. The associations between the exposure (DCDQ) and the outcomes (severe and moderate spinal pain, neck pain, mid back pain, and low back pain) were estimated by multinominal logistic regression, adjusted for all potential confounders. Stata 16 (StataCorp, College Station, Texas, USA) was used for all analyses, and significance level was set at 0.05.

## Results

**Fig. 2:**
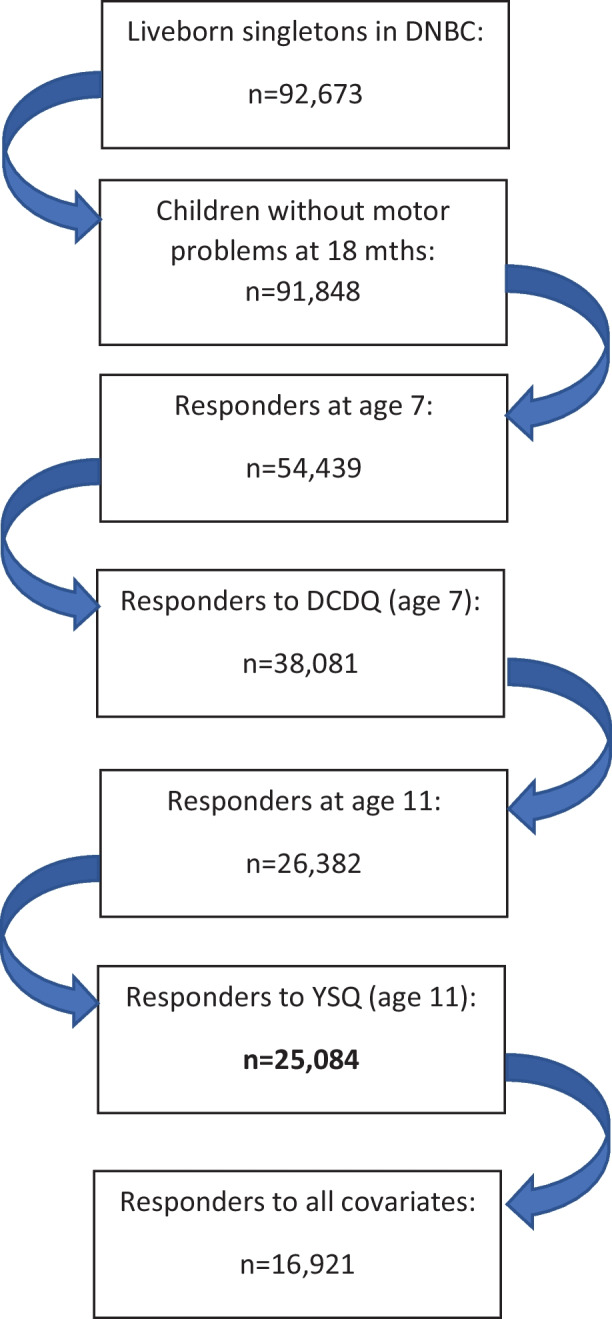
Flowchart

In total, 91,848 liveborn singletons without serious physical or mental problems were included in the DNBC between 1996 and 2003. The mothers of 54,439 children (59% of the original cohort) completed the survey when the child was 7 years of age. The 11-year follow-up was completed by 47,830 children, of which the mothers had completed the DCDQ for 26,382. Of these, 25,084 (28% of the original cohort) answered the YSQ and were included in the unadjusted analyses. To be included in the adjusted analyses, data for all covariates were required, leaving 16,921 children for these analyses (Fig. 2). Our samples differed slightly from the original cohort by being less disadvantaged on almost all parameters (i.e., higher birthweight, higher gestational age, less maternal smoking, less maternal alcohol consumption, higher education, higher income, and less spinal pain). However, all differences were very small and there were no differences in the exposure (DCDQ) (Table 1).

**Table 1:** Description of the original sample in the Banish National Birth Cohort, the sample used for raw analyses including responders to motor skills at 7 years of age and spinal pain at 11 years of age, and the sample used for adjusted analyses, including responders to all covariates as well as motor skills and spinal pain

	**All responses**	**Sample for raw analyses** ***n* = 25,184**	**Sample for adjusted analyses** ***n* = 16,921**
**Baseline variables (interviews pregnancy-18 months)**	***n* = 91,848**		
Sex, female, *n* (%)	44,723 (49)	12,943 (51)	8662 (51)
Gestational age at birth, mean weeks (SD)	40.10 (187)	40.16 (1.79)	40.24 (1.64)
Birthweight, mean, gram (SD)	3585 (570)	3605 (544)	3628 (524)
Maternal smoking during pregnancy, *n* (%)	15,157 (18)	2959 (13)	2051 (12)
Maternal alcohol consumption during pregnancy, *n* (%)	7508 (21)	4777 (20)	3460 (20)
Mother’s schooling, *n* (%)			
• Primary school only • Basic vocational training • Upper high school • Unknown	•7721 (12) • 13,253 (20) • 43,113 (65) • 1769 (3)	• 1814 (9) • 3524 (17) • 14,942 (72) • 502 (2)	• 1358 (8) • 2760 (16) • 12,406 (73) • 501 (2)
Household income, mean Danish crowns (SD)	526,426 (309,398)	540,939 (262,718)	540,843 (264,475)
**Variables from FU at age 7**	***n* = 54,439**		
DCDQ total (range 15–75), mean (SD)	67 (8)	68 (8)	68 (8)
DCDQ movement (range 6–30), mean (SD)	27 (4)	27 (4)	27 (4)
DCDQ fine motor (range 4–20), mean (SD)	18 (3)	18 (3)	18 (3)
DCDQ coordination (range 5–25), mean (SD)	22 (3)	22 (3)	22 (3)
Height age 7, mean, cm (SD)	126 (6)	126 (6)	126 (6)
Weight age 7, mean, kg (SD)	25 (5)	25 (4)	25 (4)
**Variables from FU at age 11**	***n* = 47,830**		
Moderate spinal pain, *n* (%)	13,441 (30)	7188 (29)	4854 (29)
Severe spinal pain, *n* (%)	5438 (12)	2755 (11)	1847 (11)
Moderate neck pain, *n* (%)	11,127 (25)	5984 (24)	4026 (24)
Severe neck pain, *n* (%)	3403 (8))	1778 (7)	1194 (7)
Moderate thoracic pain, *n* (%)	6225 (14)	3179 (13)	2149 (13)
Severe thoracic pain, *n* (%)	1928 (4)	947 (4)	624 (4)
Moderate lumbar pain, *n* (%)	4711 (10)	2342 (9)	1586 (9)
Severe lumbar pain, *n* (%)	1687 (4)	785 (3)	540 (3)

*DCDQ* Developmental Coordination Disorder Questionnaire, *SD* standard deviation, *FU* follow-up

Almost one-third (29%) reported moderate spinal pain, and 11% severe spinal pain. The most common pain region was the neck (both moderate and severe), and for all regions, the prevalence was about three times higher for moderate pain than for severe pain (Table 1).

**Fig. 3:**
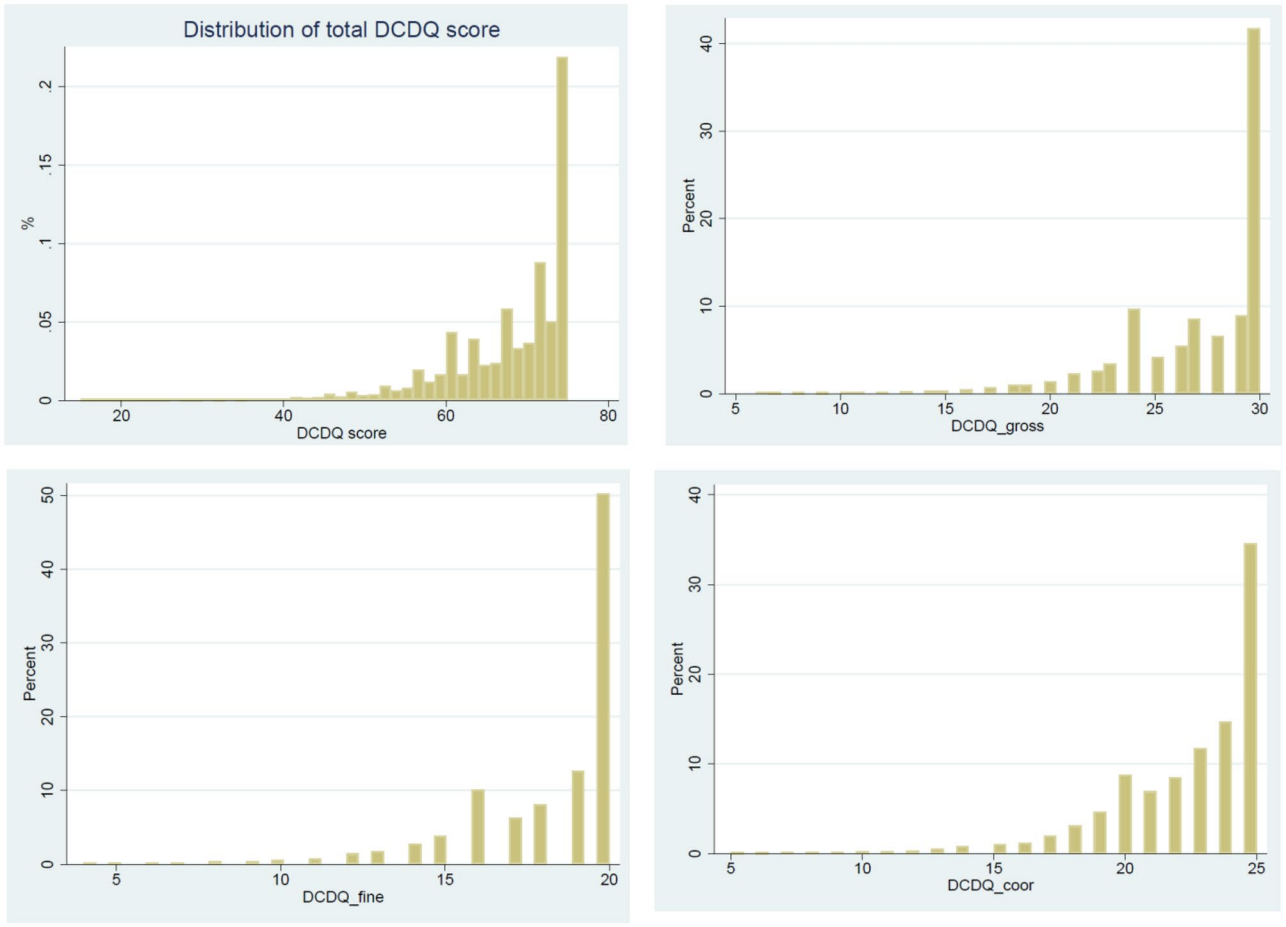
Distribution of DCDQ scores for the total score, gross motor skills, fine motor skills, and coordination at age 7. DCDQ: Developmental Coordination Disorder Questionnaire at the age of seven years; score: total score; gross: subscale for gross motor skills; fine: subscale for fine motor skills; coor: subscale for coordination

DCDQ scores were strongly skewed (Fig. 3). The tenth percentile (cut point between category 3 and 2) was 56 for the total DCDQ score, 21 for the gross motor skills subscale, 14 for the fine motor skill subscale, and 17 for the coordination subscale.

### Association with spinal pain

**Fig. 4:**
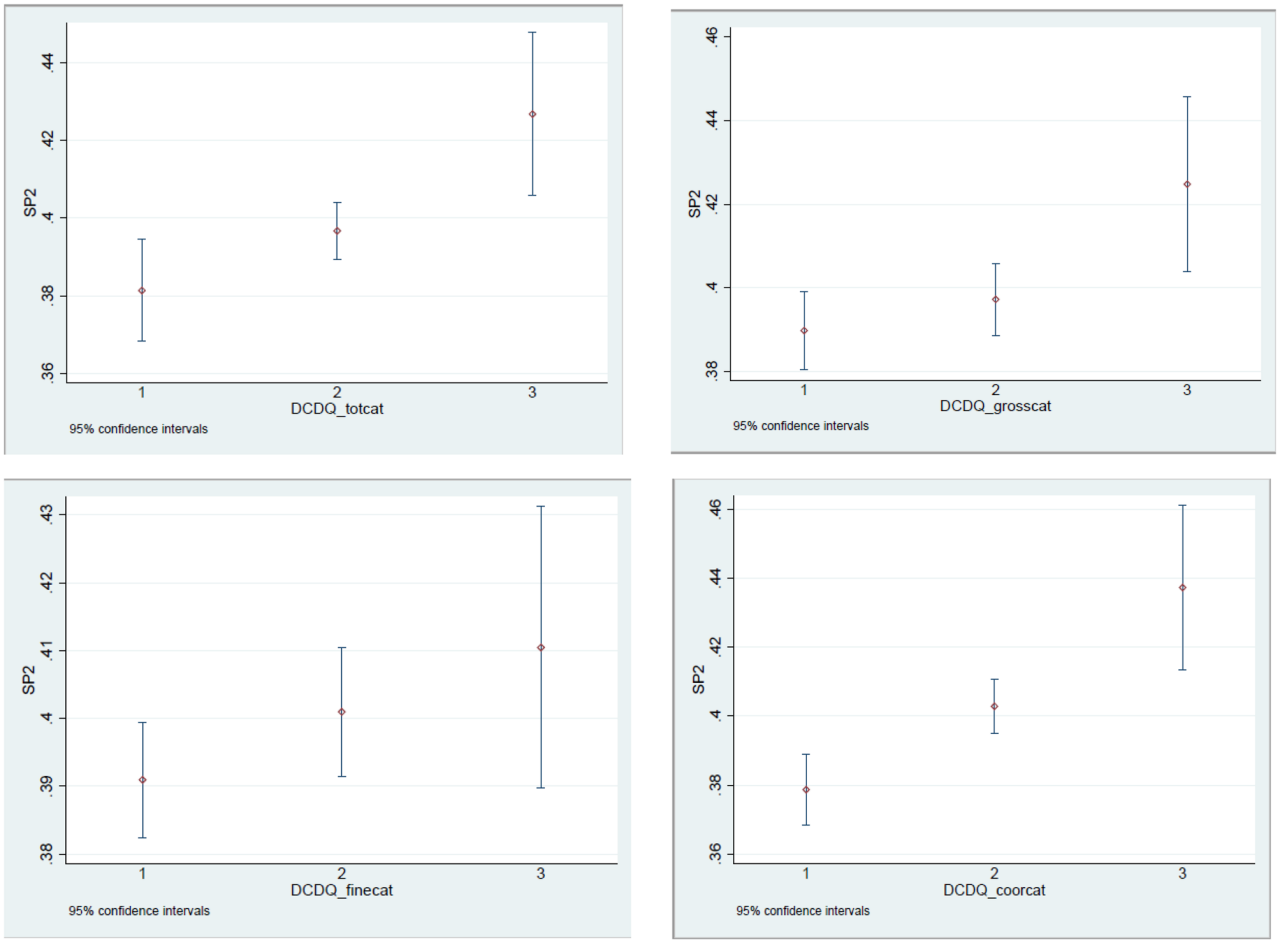
Prevalence of spinal pain (moderate and severe combined) at age 11 by categories* of motor skills at age 7; 95% confidence interval. *1: maximum score, 2: above the tenth percentile but below maximum score; 3: the lowest 10 percent. SP2: moderate and severe spinal pain combined; DCDQ: Developmental Coordination Disorder Questionnaire at the age of seven; totcat: categories for total score; grosscat: categories for subscale for gross motor skills; finecat: categories for subscale for fine motor skills; coorcat: categories for subscale for coordination

The graphs in Fig. 4 generally show an increasing frequency of spinal pain at age 11 with increasing motor difficulties at age 7, but the differences are small (please note the scale on the y-axis). Results were similar for neck pain and mid back pain, whereas no relationship was seen with low back pain. Graphs by region can be seen in Supplementary Material 2.

There were slightly increased relative risk ratios (RRR) for moderate spinal pain, neck pain, and mid back pain at age 11 with lower DCDQ scores at age 7. The relationship was consistent but weak with RRR ranging from 1.07 to 1.22 for children with the lowest levels of motor skills and was generally not statistically significant. There was no association with low back pain (Table 2).

**Table 2:** Adjusted relative risk ratios for developing moderate spinal pain at age 11 (95% confidence intervals) dependent on motor development measures

	Moderate SP (*n* = 7110)	Moderate NP (*n* = 5895)	Moderate MBP (*n* = 3223)	Moderate LBP (*n* = 2390)
**DCDQ total**				
* Best (n* = *4007)* * Medium(n* = *13,073)* * Poorest (n* = *1701)*	Ref 1.02 (0.93–1.12) 1.12 (0.97–1.30)	Ref 0.98 (0.89–1.07) 1.07 (0.91–1.24)	Ref 1.04 (0.92–1.17) 1.19 (0.98–1.44)	Ref 1.08 (0.94–1.25) 1.07 (0.85–1.35)
**DCDQ gross**				
* Best (n* = *7933)* * Medium (n* = *9405)* * Poorest (n* = *1678)*	Ref 0.99 (0.92–1.07) 1.09 (0.95–1.25)	Ref 0.96 (0.89–1.04) 1.10 (0.96–1.27)	Ref 0.97 (0.88–1.07) 1.08 (0.90–1.29)	Ref 1.03 (0.92–1.16) 0.90 (0.73–1.12)
**DCDQ fine**				
* Best (n* = *9595)* * Medium (n* = *7834)* * Poorest (n* = *1680)*	Ref 1.04 (0.96–1.12) 1.13 (0.98–1.29)	Ref (0.93–1.10) 1.08 (0.93–1.25)	Ref 1,01 (0.91–1.12) 1.21 (1.01–1.45)*	Ref (0.91–1.16) 1.23 (1.00–1.51)*
**DCDQ coordination**				
* Best (n* = *6586)* * Medium(n* = *11,123)* * Poorest (n* = *1319)*	Ref 1.08 (1.00–1.17)* 1.21 (1.04–1.42)*	Ref 1.08 (0.99–1.17) 1.16 (0.99–1.37)	Ref 1.09 (0.98–1.21) 1.22 (1.00–1.50)*	Ref 1.10 (0.98–1.25) 1.09 (0.86–1.38)

All relative risk ratios adjusted for sex, birth weight, child height and weight at age seven, maternal smoking during pregnancy, maternal alcohol consumption during pregnancy, household income, and mother’s schooling

*DCDQ* Developmental Coordination Disorder Questionnaire with total score and three subscales: control of movement (gross), fine motor function (fine), and coordination; *SP* spinal pain; *NP* neck pain; *MBP* mid back pain; *LBP* low back pain. Poorest: the lowest 10% of the sample, Medium: above the 10^th^ percentile but not maximum score, Best: maximum score

*p < 0.05

The associations were stronger for severe spinal pain than for moderate pain. There was consistently increased RRRs for spinal pain, neck pain, and mid back pain, with decreasing DCDQ scores. The associations ranged from 1.04 to 1.87 and were statistically significant in 19 of the 24 estimated associations. There were no associations with low back pain (Table 3).

**Table 3:** Adjusted relative risk ratios for developing severe spinal pain at age 11 (95% confidence intervals) dependent on motor development measures

	Severe SP (*n* = 2768)	Severe NP (*n* = 1734)	Severe MBP (*n* = 958)	Severe LBP (*n* = 806)
**DCDQ total**				
* Best (n* = *4007)* * Medium(n* = *13,073)* * Poorest (n* = *1701)*	Ref 1.19 (1.04–1.37)* 1.54 (1.25–1.91)*	Ref 1.23 (1.04–1.45)* 1.61 (1.26–2.08)*	Ref 1.30 (1.03–1.63)* 1.61 (1.14–2.26)*	Ref 1.04 (0.83–1.33) 1.24 (0.85–1.82)
**DCDQ gross**				
* Best (n* = *7933)* * Medium (n* = *9405)* * Poorest (n* = *1678)*	Ref 1.04 (0.93–1.17) 1.20 (0.99–1.46)	Ref 1.05 (0.92–1.20) 1.19 (0.95–1.50)	Ref 1.22 (1.02–1.47)* 1.27 (0.93–1.73)	Ref 0.88 (0.72–1.07) 0.97 (0.69–1.38)
**DCDQ fine**				
* Best (n* = *9595)* * Medium (n* = *7834)* * Poorest (n* = *1680)*	Ref 1.18 (1.06–1.32)* 1.31 (1.07–1.60)*	Ref 1.18 (1.03–1.35)* 1.28 (1.00–1.62)*	Ref 1.27 (1.06–1.52)* 1.60 (1.18–1.52)*	Ref 1.03 (0.84–1.26) 1.07 (0.73–1.57)
**DCDQ coordination**				
* Best (n* = *6586)* * Medium(n* = *11,123)* * Poorest (n* = *1319)*	Ref 1.25 (1.11–1.40)* 1.53 (1.23–1.91)*	Ref 1.26 (1.10–1.45)* 1.59 (1.23–2.05)*	Ref 1.40 (1.15–1.69)* 1.87 (1.34–2.61)*	Ref (0.84–1.27) 0.93 (0.60–1.45)

All relative risk ratios adjusted for sex, birth weight, child height and weight at age seven, maternal smoking during pregnancy, maternal alcohol consumption during pregnancy, household income, and mother’s schooling

*DCDQ* Developmental Coordination Disorder Questionnaire with total score and three subscales: control of movement (gross), fine motor function (fine), and coordination; *SP* spinal pain; *NP* neck pain; *MBP* mid back pain; *LBP* low back pain. Poorest: the lowest 10% of the sample, Medium: above the 10th percentile but not maximum score, Best: maximum score

*p < 0.05

The relationship was driven by the subscales for fine motor skills and coordination with no or weak associations for gross motor skills. The highest estimated relative risk ratio was 1.87 (95% CI:1.34–2.61) for development of severe mid back pain in the group with poorest coordination (Tables 2 and 3).

## Discussion

We found a consistent pattern of more reporting of neck or mid back pain at age 11 with poorer motor skills scores at age 7. The relationship was stronger for severe than for moderate pain, but all estimates were small and only statistically significant for severe pain. There was no relationship with low back pain.

According to the recent review by Noll et al. [41], only two studies have investigated the influence of balance on back pain in children and adolescents: a prospective study of balance at age 16 and back pain at age 34, and a cross-sectional study of balance and back pain the past week in children aged 8–12 years. Neither of the two found an association, but as one did not report paediatric spinal pain and the other was cross-sectional, results are difficult to relate to the present study. To our knowledge, other domains of motor skills have not been investigated in relation to spinal pain in children.

A major strength of this study is the large general population-based sample. Almost one-third of all pregnant women in Denmark in the years from 1996 to 2002 were enrolled in the DNBC cohort (29) and 55% of the invited children responded to the 11-year follow-up. Nevertheless, attrition is substantial, and pregnant women who participated in the DNBC were generally healthier and had higher socioeconomic status than women who did not participate [42]. The differential drop out pattern could induce participation bias, as children of parents with lower socioeconomic status and shorter education have higher incidence of back pain [43, 44]. However, although only 28% of the original cohort is included in the final analyses, we believe the possible attrition bias is limited since adding confounders to the model did not change the estimates (unadjusted estimates not shown) and the prevalence of spinal pain was similar to those found in another Danish study investigating the same age group with the same instrument (YSQ) but with response rates above 90% [35]. Furthermore, Greene et al. found minimal influence of attrition bias on selected associations in the DNBC cohort [45] and prevalence of spinal pain with inverse probability weighting to resemble the general Danish population has shown similar rates as the final sample in this study [34].

The self-reported measure of back pain could create some concern. However, although back pain in childhood was previously considered rare and a sign of serious underlying pathology, more recent studies have indicated that the condition is common, and it is usually not possible to diagnose a specific patho-anatomical cause for the pain, suggesting that the vast majority of spinal pain is non-specific. In that light, the self-reports of pain are probably the best option.

The most important limitation of this study is the measurements of motor skills. The DCDQ was originally developed to detect children with motor skills deficiencies [32] and is therefore better to differentiate among poor performers than across a normal range of children. This was illustrated by high sensitivity demonstrated in clinical samples but not in population-based samples [30] and was also clear by the strong ceiling effect in our sample with more than 80% of mothers answering positively to most questions.

Our results should trigger more research into the area of motor skills as a potential vehicle for prevention of development of spinal pain in the young population. However, future studies should use instruments developed to describe motor skills in normal populations. Furthermore, the prevalence of spinal pain is still low at age 11 compared to older adolescents, especially for low back pain which represents the largest burden in adults, and therefore investigations with longer follow-up are needed.

## Conclusion

Our results indicate a link between motor development as measured by the DCDQ at 7 years of age and neck and mid back pain at 11 years of age. The relationship was driven by the subscales of coordination and fine motor skills, whereas associations with “control of movement” were weak or non-existent. No associations with low back pain were detected. Thus, improvement of motor skills in young children might reduce the future burden of neck and mid back pain and should be a target of future research.

## Supplementary Information

Below is the link to the electronic supplementary material.

Supplementary file1 (DOCX 18 KB)

Supplementary file2 (DOCX 381 KB)

## Abbreviations

DNBC: The Danish National Birth Cohort
DCDQ: Developmental Coordination Disorder Questionnaire
rFPS: Revised Faces Pain Scale
RRR: Relative risk ratio
SP: Spinal pain (neck pain, mid back pain and/or low back pain)
YSQ: The Young Spine Questionnaire
CI: Confidence intervals

## Declarations

### Disclaimer

The funder/sponsor did not participate in the work.
