# Supplementary material for: Motor skills at 7 years of age and spinal pain at 11 years of age: a cohort study of 26,000 preadolescents
Source: Eur J Pediatr. 2023 Apr 12;182(6):2843–53. doi: 10.1007/s00431-023-04964-8 (PMC10257602; doi:10.1007/s00431-023-04964-8)
Supplement: Supplementary file 1 — Supplementary file1 (DOCX 18 KB) [file 431_2023_4964_MOESM1_ESM.docx]

**Supplementary material 1** (METHODS)

**Selected questions and answer options from an interview during pregnancy (approximately gestational age of 30 weeks)**

B089. During all your time as pregnant – also in the very beginning – how many times do you think that you have had 5 drinks or more in one evening or in one event?

1. Never

2. >1time

B074. Have you smoked since the last interview?

1. Yes

2. No

**Selected questions and answer options from the interview when the child was approximately 18 months old:**

D152. At which level did you complete your schooling?

The 20 response options were categorized into four levels: ‘Primary school only’, ‘Basic vocational training’, ‘Upper high school’ and ‘Unknown’.

D161A. What is the yearly income before tax for the family? __________ DKK (0-99.999.999)

Responses were categorized into ‘<300000’, ‘300000-399999’, ‘400000-499999’, ‘500000-599999’, ‘600000-699999’, ‘700000-999999’ and ’>999999’.

**Selected questions and answer options from the questionnaire when the child was approximately 7 years old (completed by the mother):**

*The Developmental Coordination Disorder Questionnaire:*

Do you find the following statements true of your son, when you compare him with other children of

the same age?

1. Your child *throws a ball* in the same controlled and accurate fashion as other children the same

age.

2. Your child *catches* a small *ball* (e.g., tennis ball size) just as well as other children when it is

thrown from a distance of 6 to 8 feet.

3. Your child *hits* an approaching *ball* or *birdie* with a bat or racquet as accurately as other children the

same age.

4. Your child *jumps* as easily *over* obstacles found in garden or play environment as other children

the same age.

5. Your child *runs* as fast and in a *similar* way to other children of the same sex and age

6. Your child can control his movements just as well as other children of the same age, and accomplish

what he sets out to do. For example, when he wants to build a hideout (with cardboard boxes or cushions etc.), a tower of blocks or other toys or when he moves around playground equipment.

7. Your child can draw and write just as quickly as the other children in the class.

8. Your child’s printing or *writing* letters, numbers and words is *legible*, precise and accurate or, if

your child is not yet printing, *colors and draws* in a coordinated way, and draws things that you can

recognize.

9. Your child uses appropriate *effort* or tension when drawing, printing or writing (no excessive *pressure*

or tightness of grasp, writing not too heavy or dark, or too light).

10. Your child *cuts* out pictures and *shapes* as accurately and easily as other children the same age.

11. Your child learns *new motor tasks* (e.g., swimming, rollerblading) easily and does not require

more practice or time than others to achieve the same level of skill

12. Your child can easily move around without bumping in to and upsetting/breaking things. He is not

clumsier than other children his age.

13. Your child is as *quick and good at* tidying up, putting on shoes, tying shoes, dressing, etc. as other

children the same age.

14. Your child is interested in and *likes* participating in *sports or active* games requiring good motor

skills.

15. Your child can sit for as long periods of time as other children the same age, without getting tired,

*slouching* or “falling” off the chair.

All 15 questions could be scored as: 1 ‘not true’, 2 ‘a little true’, 3 ‘fairly true’, 4 ‘true’, 5 ‘very true’.

*Other*

Height: __________cm

Weight: __________kg

**Selected questions and answer options from the questionnaire when the child was approximately 11 years old (completed by the child):**

*Spinal pain*

*The next questions are about pain in your neck and back.*

E089 Have you suffered from neck pain?

1. yes, often

2. yes, from time to time

3. yes, once or twice

4. never -> E090

99. not answered

*(E089A:Question may be skipped without answering)*

Dependent on E089

E089A How much did it hurt at its worst? 1 is "not at all" and 6 is "really very much”.

1-6, not answered=99, not applicable=100

E090 Have you suffered from middle back pain?

1. yes, often

2. yes, from time to time

3. yes, once or twice

4. never -> E092

99. not answered

*(E090A:Question may be skipped without answering)*

Dependent on E090

E090A How much did it hurt at its worst? 1 is "not at all" and 6 is "really very much”.

1-6, not answered=99, not applicable=100

E091 Have you suffered from low back pain?

1. yes, often

2. yes, from time to time

3. yes, once or twice

4. never -> E093

99. not answered

*(E091A:Question may be skipped without answering)*

Dependent on E091

E091A How much did it hurt at its worst? 1 is "not at all" and 6 is "really very much”.

1-6, not answered=99, not applicable=100
