# Supplementary material for: Motor skills at 7 years of age and spinal pain at 11 years of age: a cohort study of 26,000 preadolescents
Source: Eur J Pediatr. 2023 Apr 12;182(6):2843–53. doi: 10.1007/s00431-023-04964-8 (PMC10257602; doi:10.1007/s00431-023-04964-8)
Supplement: Supplementary file 2 — Supplementary file2 (DOCX 381 KB) [file 431_2023_4964_MOESM2_ESM.docx]

**Supplementary material 2** (RESULTS)

**Figure S1. Prevalence of neck pain (moderate and severe combined) at age 11 by early motor skills groups***


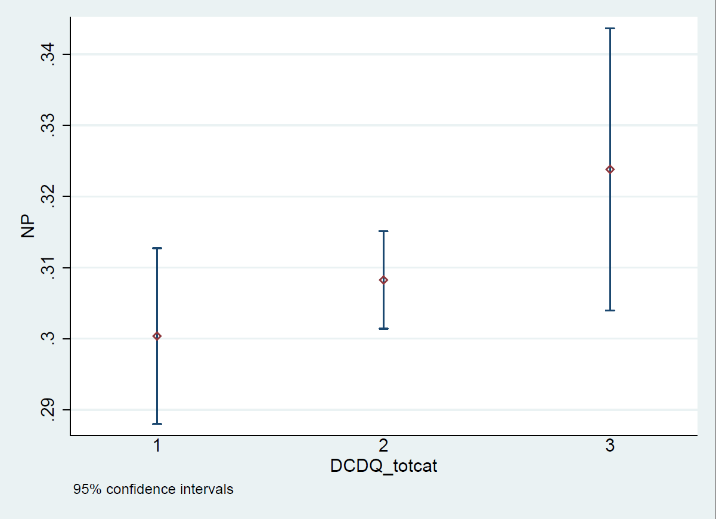

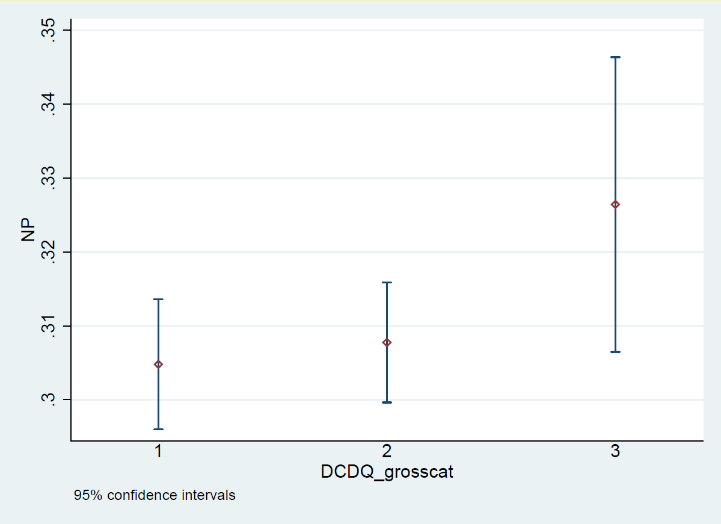


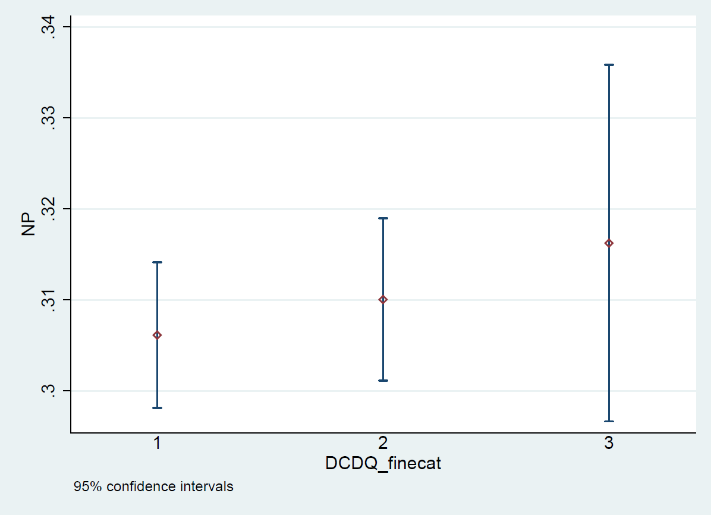

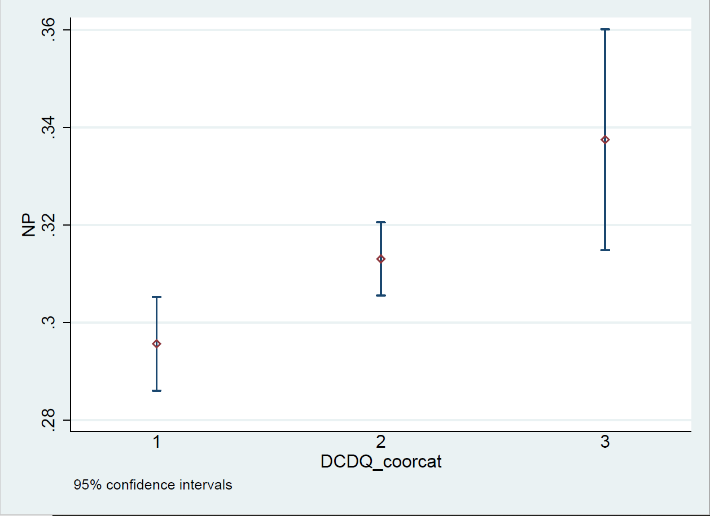


NP: neck pain (moderate and severe combined), DCDQ: Developmental Coordination Disorder Questionnaire at the age of seven; totcat: total score category; grosscat: categories for subscale for gross motor skills; finecat: categories for subscale for fine motor skills; coorcat: categories for subscale for coordination.

*1: maximum score, 2: above the tenth percentile but below maximum score; 3: the lowest 10 percent

**Figure S2. Prevalence of mid back pain (moderate and severe combined) at age 11 by early motor skills groups***


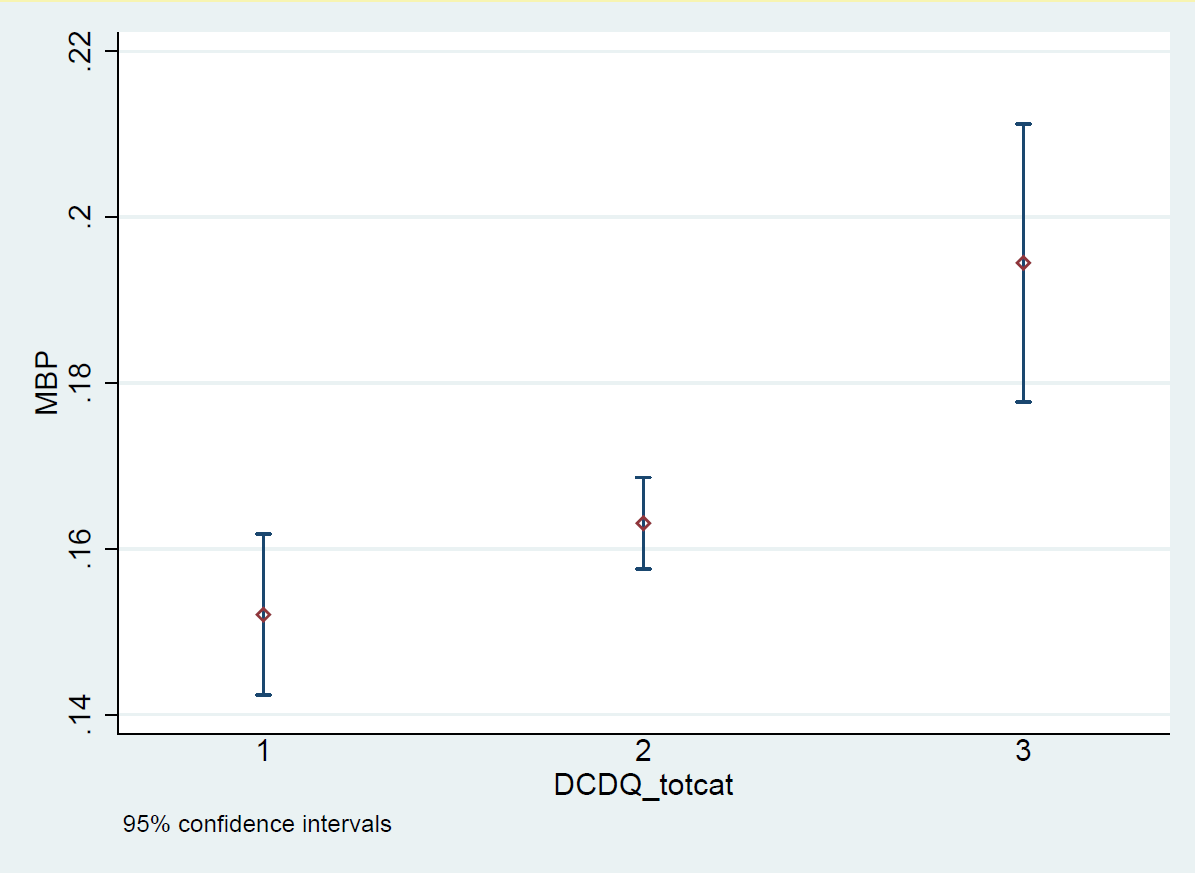

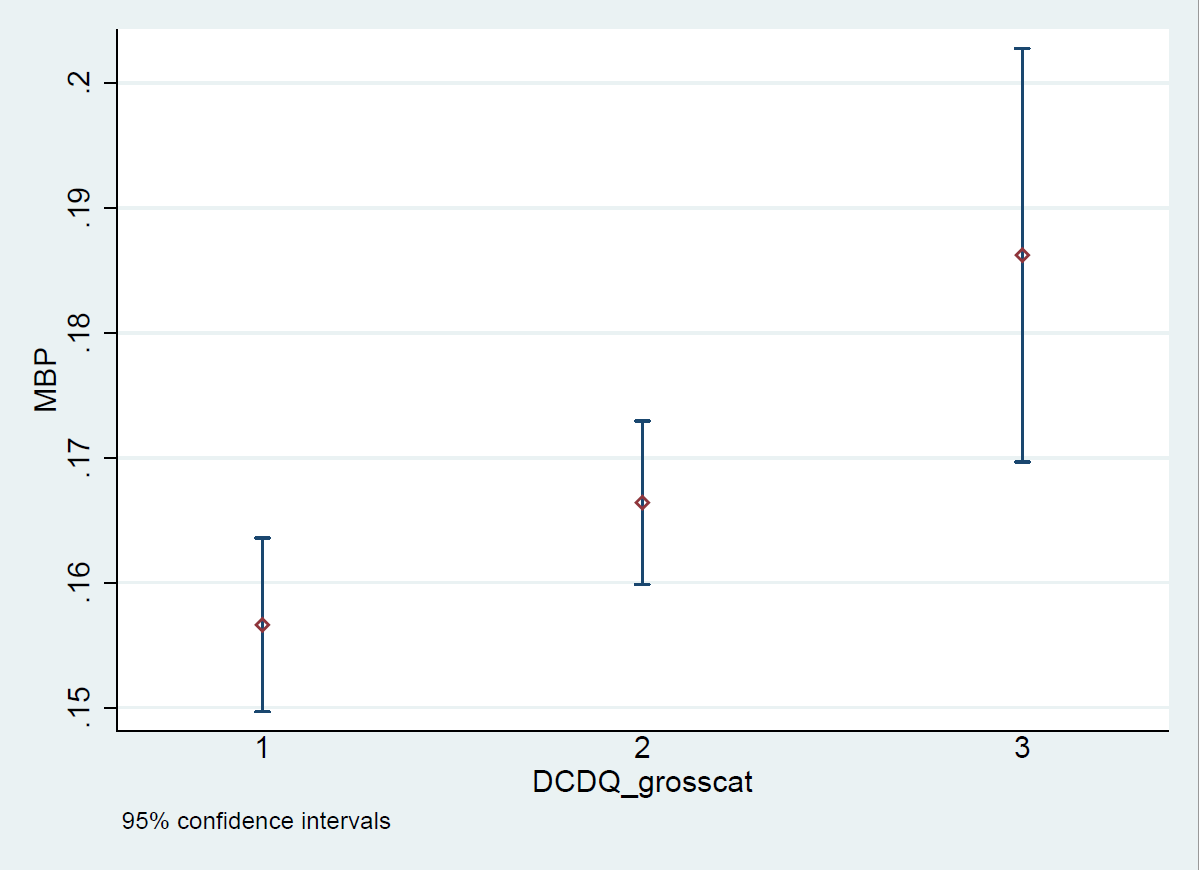


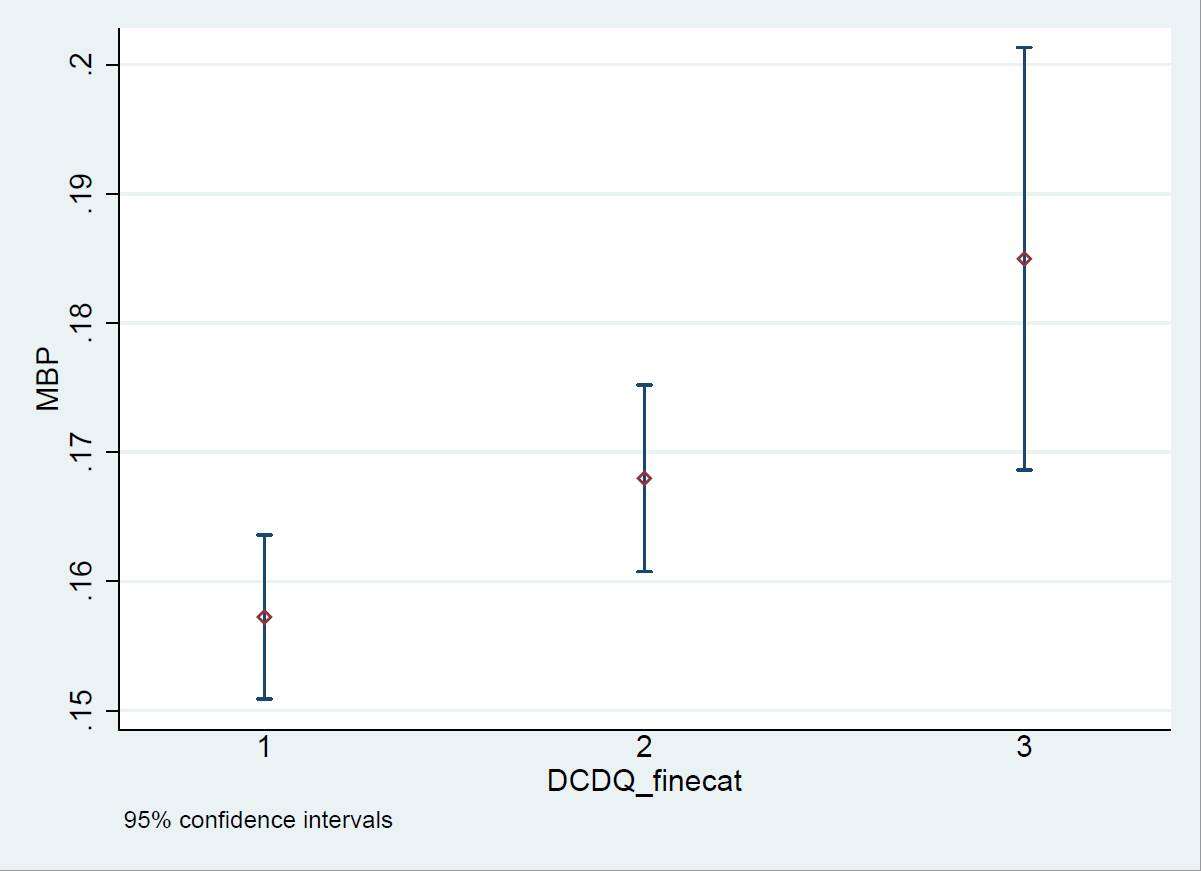

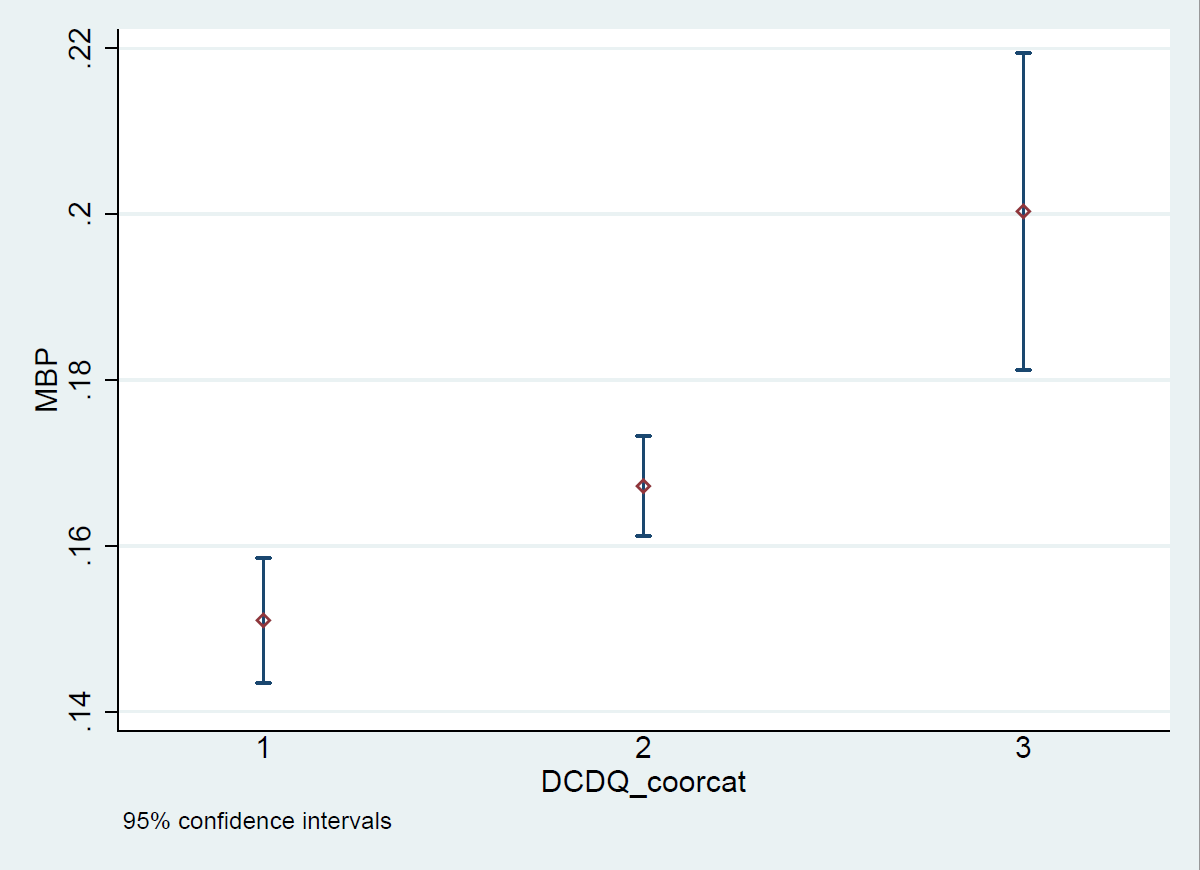


MBP: mid back pain (moderate and severe combined), DCDQ: Developmental Coordination Disorder Questionnaire at the age of seven; totcat: total score category; grosscat: categories for subscale for gross motor skills; finecat: categories for subscale for fine motor skills; coorcat: categories for subscale for coordination.

*1: maximum score, 2: above the tenth percentile but below maximum score; 3: the lowest 10 percent

**Figure S3. Prevalence of low back pain (moderate and severe combined) at age 11 by early motor skills groups***


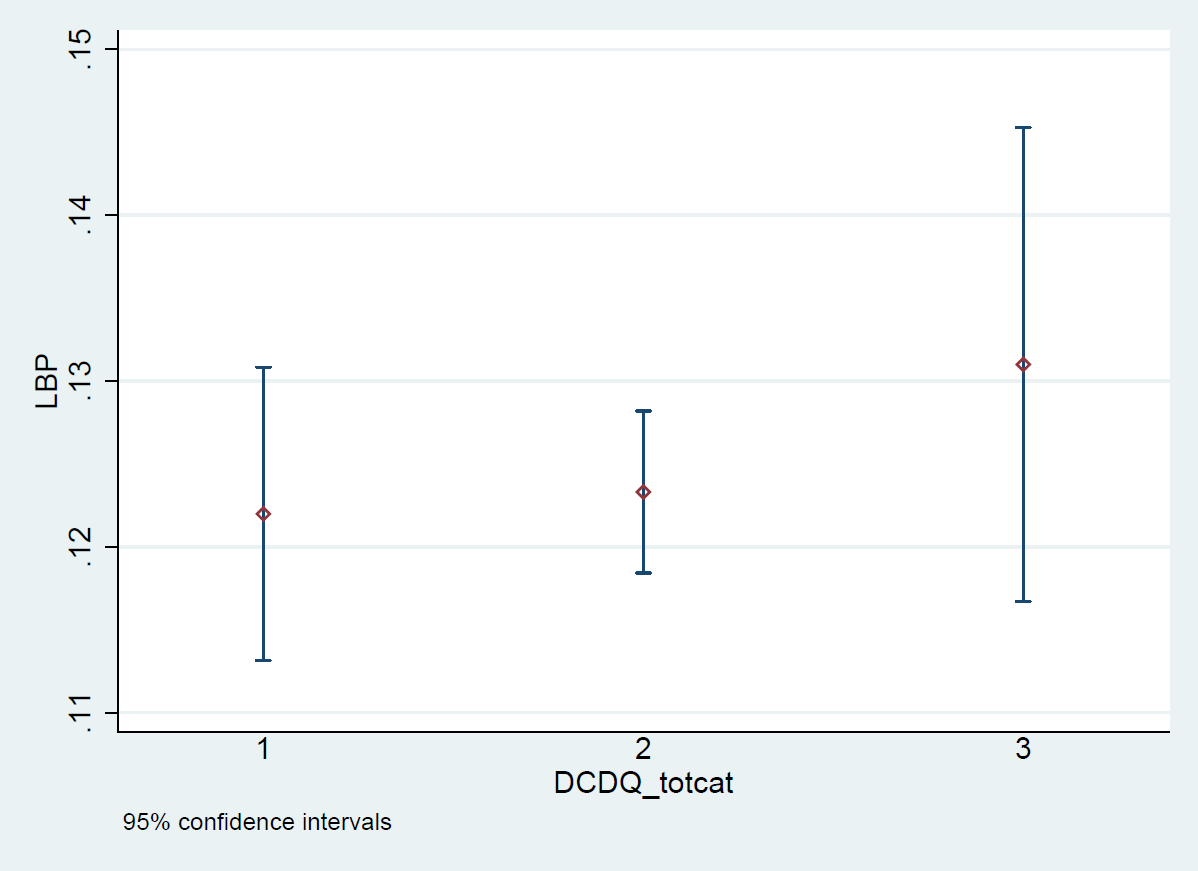

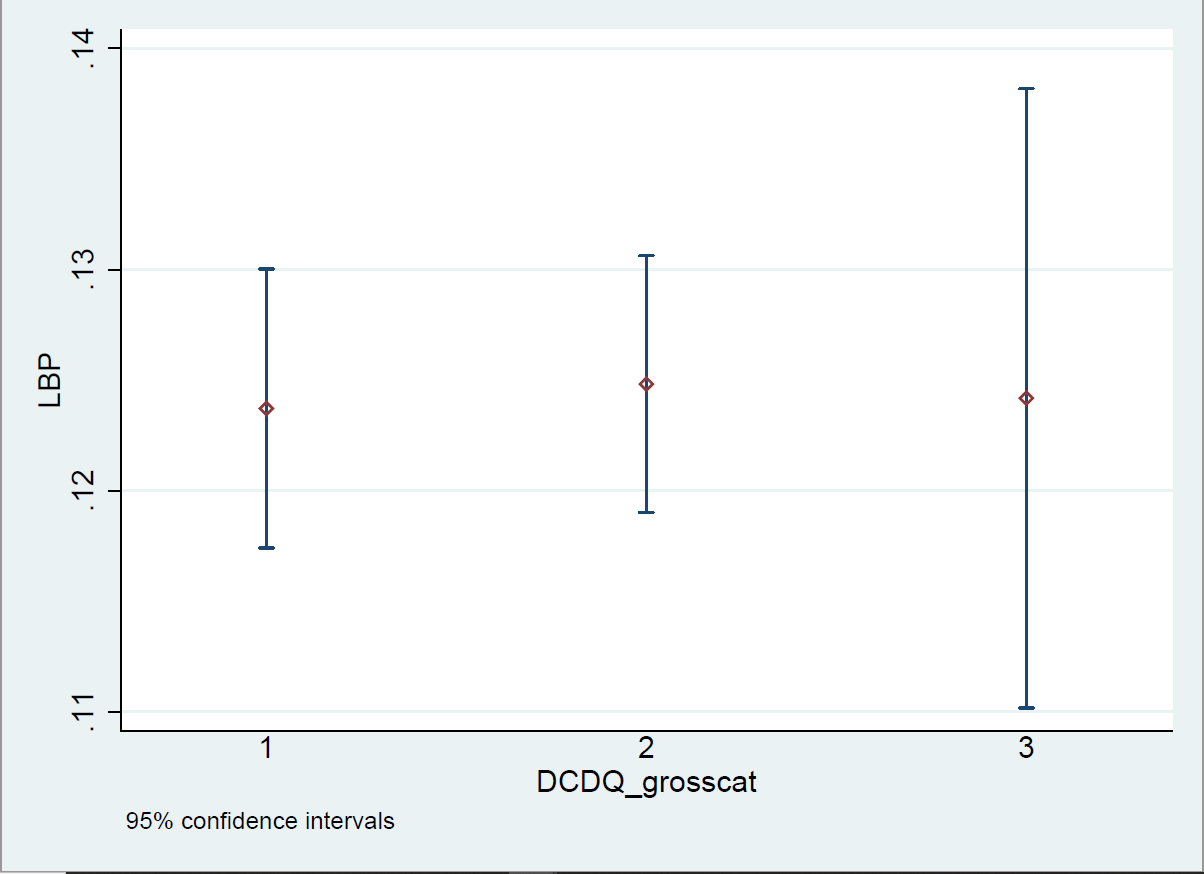


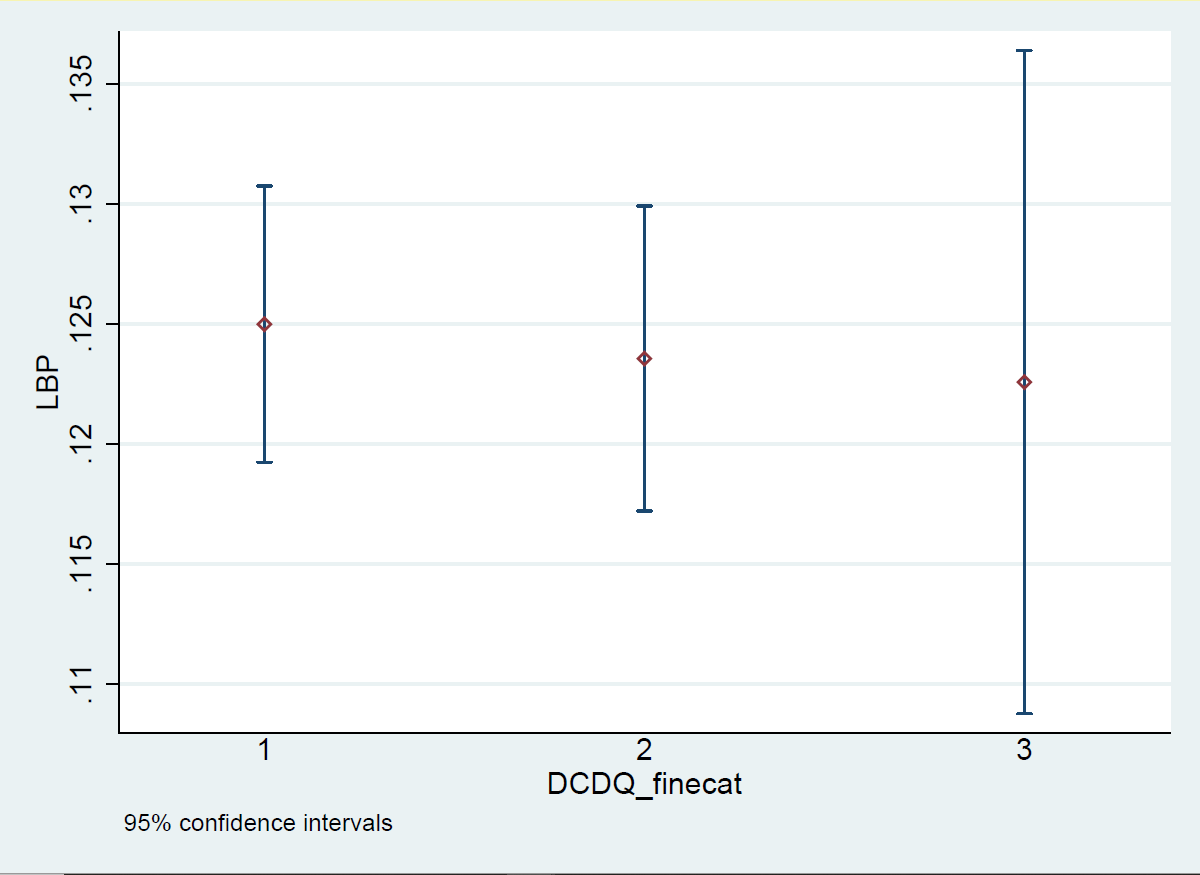

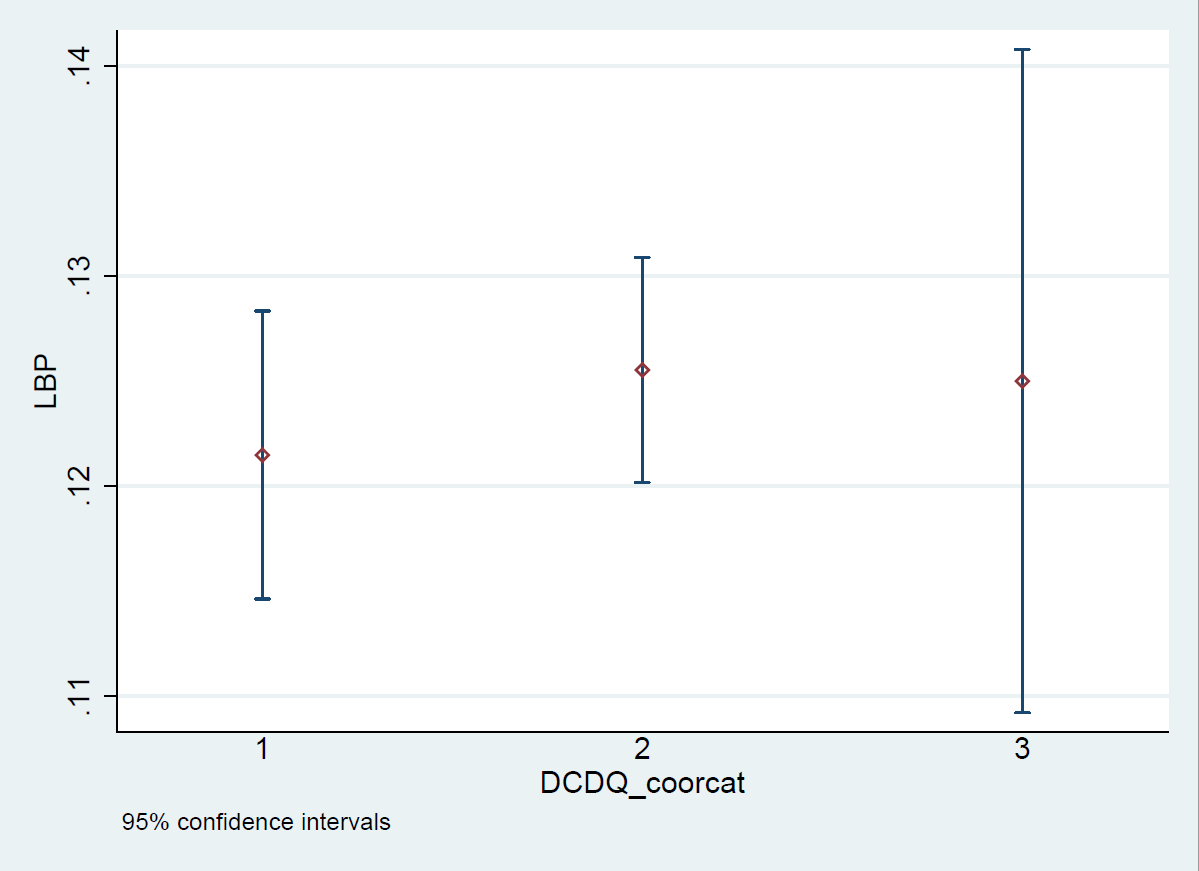


LBP: low back pain (moderate and severe combined), DCDQ: Developmental Coordination Disorder Questionnaire at the age of seven; totcat: total score category; grosscat: categories for subscale for gross motor skills; finecat: categories for subscale for fine motor skills; coorcat: categories for subscale for coordination.

*1: maximum score, 2: above the tenth percentile but below maximum score; 3: the lowest 10 percent
